# Occupational cold exposure is associated with increased reporting of airway symptoms

**DOI:** 10.1007/s00420-021-01694-y

**Published:** 2021-04-17

**Authors:** Albin Stjernbrandt, Nikolai Stenfors, Ingrid Liljelind

**Affiliations:** 1 Section of Sustainable Health, Department of Public Health and Clinical Medicine, Umeå University, 90187 Umeå, Sweden; 2 Section of Medicine, Department of Public Health and Clinical Medicine, Umeå University, 90187 Umeå, Sweden

**Keywords:** Asthma, Chronic obstructive pulmonary disease, Cough, Cold exposure, Occupational exposure, Sweden

## Abstract

**Objective:**

To determine if exposure to cold environments, during work or leisure time, was associated with increased reporting of airway symptoms in the general population of northern Sweden.

**Methods:**

Through a population-based postal survey responded to by 12627 subjects, ages 18–70, living in northern Sweden, the occurrence of airway symptoms was investigated. Cold exposure during work or leisure time was self-reported on numerical rating scales. Binary logistic regression was used to determine the statistical association between cold exposure and airway symptoms.

**Results:**

For currently working subjects (*N* = 8740), reporting any occupational cold exposure was associated to wheeze (OR 1.3; 95% CI 1.1–1.4); chronic cough (OR 1.2; 95% CI 1.1–1.4); and productive cough (OR 1.3; 95% CI 1.1–1.4), after adjusting for gender, age, body mass index, daily smoking, asthma, and chronic obstructive pulmonary disease. Leisure-time cold exposure was not significantly associated to reporting airway symptoms.

**Conclusions:**

Occupational cold exposure was an independent predictor of airway symptoms in northern Sweden. Therefore, a structured risk assessment regarding cold exposure could be considered for inclusion in the Swedish workplace legislation.

**Supplementary Information:**

The online version contains supplementary material available at 10.1007/s00420-021-01694-y.

## Introduction

Around 4 million people live in the Arctic region (Larsen and Fondahl 2014) and are subjected to a cold climate. Exposure to cold air is associated with increased morbidity and mortality (Rocklöv and Forsberg 2008), especially among the elderly and those with cardiorespiratory disease (Analitis et al. 2008; Hajat et al. 2007; Näyhä 2005; Schwartz 2005; The Eurowinter Group 1997). Cold-related cardiorespiratory symptoms in turn predict a future higher occurrence of hospital admissions and mortality (Ikäheimo et al. 2020). Exposure to cold and dry air is also associated with increased frequency of airway infections (Mäkinen and Hassi 2009), possibly due to increased bacterial survival (Handley and Webster 1995) and inhibition of epithelial defense mechanisms (Eccles 2002). It has been reported that 26% of men and 31% of women experience cold-related airway symptoms: increased mucus production may occur already at − 7 °C, whereas onset of cough, wheezing and dyspnea has been reported at − 18 °C or colder (Harju et al. 2010). Up to one-third of subjects with allergic rhinoconjunctivitis and asthma avoid outdoor activities during cold spells, to prevent worsening of symptoms (Millqvist et al. 1987). Also, low outdoor temperatures are associated with increased respiratory symptoms, increased rescue inhaler use and decreased lung function in patients with chronic obstructive pulmonary disease (COPD) (McCormack et al. 2017).

A large epidemiological study in Finland reported that wheeze, tightness of breath, cough, and sputum were more prevalent in areas with cold climate, and the highest prevalence of chronic bronchitis was found among smoking outdoor workers (Kotaniemi et al. 2002). Studies on Inuit in the Canadian arctic have described COPD suggested to be accelerated by prolonged ventilation of cold air (Rode and Shephard 1994; Schaefer et al. 1980). Winter sport endurance athletes, such as cross-country skiers, are repeatedly exposed to cold air during prolonged periods of exercise, and show an increased prevalence of airway symptoms, bronchial hyperreactivity, and asthma (Carlsen et al. 2008). The high prevalence of asthma among elite athletes is believed to be related to inhalation of cold and dry air, leading to desiccation of the airways, which induces a hyperosmolar milieu in the airway mucosa. If the exposure is prolonged and repeated, airway epithelial damage, inflammation, and bronchial hyperreactivity may develop. While some authors argue that cold air is only a trigger for airway symptoms (Koskela 2007), others state that it might actually cause airway dysfunction (Giesbrecht 1995; Kotaniemi et al. 2003). To the authors’ knowledge, there have been no previous population-based studies on cold-related respiratory symptoms in northern Sweden, though ambient cold exposure is often profound in this region.

The aim of the present study was to determine if exposure to cold environments, during work or leisure time, was associated with increased reporting of airway symptoms in the general population of northern Sweden.

## Materials and methods

### Study design and setting

This cross-sectional, population-based study was part of a research project called Cold and Health in Northern Sweden (CHINS), which was launched in 2015 to broadly investigate adverse health effects from cold climate exposure in the four northernmost counties of Sweden: Norrbotten, Västerbotten, Västernorrland, and Jämtland. The data collection was performed by means of a postal survey on a sample of men and women between 18 and 70 years of age, living in the study area, who were drawn from the national Swedish population register. The survey collected information regarding anthropometry, general and airway symptoms, tobacco habits, and occupation. The data collection has previously been described in detail (Stjernbrandt et al. 2017). The mean monthly temperature during the study period spanned from about − 9 to 5 °C (Swedish Metrological and Hydrological Institute 2015).

### Variables and statistical methods

Since continuous variables were not normally distributed, data were described as median values and interquartile ranges (IQR), while categorical variables were presented as numbers and percentages. Dichotomous dependent variables were: *wheeze* (“Have you experienced wheezing in the chest at any time during the last twelve months?”); *chronic cough* (“Have you suffered from long-standing cough during the past few years?”); and *productive cough* (”Do you usually cough up mucus, or have you had mucus in your chest that you have had a hard time clearing?”). *Occupational* or *leisure-time cold exposur*e were assessed by two questionnaire items:

1. “During work I am exposed to outdoor or cold environments.”
2. “During leisure time I am exposed to outdoor or cold environments.”

The answers were given on whole number numerical rating scales (NRS), ranging from one (“do not agree”) to ten (“fully agree”). Additional independent variables used for adjusting were: gender (male/female), age (years); body mass index (BMI; kg/m^2^); daily smoking (yes/no); physician-diagnosed asthma (yes/no); and physician-diagnosed COPD (yes/no). Age was subsequently categorized by quartiles, cold exposure based on the 50th percentile, and BMI by clinically used thresholds for under- and overweight. Current occupation was coded in accordance with the International Standard Classification of Occupations (International Labour Organization 2012). Binary logistic regression was used for simple and multiple analyses. A *p* value < 0.05 was considered statistically significant. Statistical analyses were performed using SPSS (version 26.0, IBM Corporation, Armonk, NY, USA).

## Results

### Recruitment

The study population consisted of 12627 subjects (response rate 35.9%). A previously published non-responder analysis based on the sampling frame showed that the response rate was higher among women, and increased with age (Stjernbrandt et al. 2017).

### Descriptive data

**Fig. 1:**
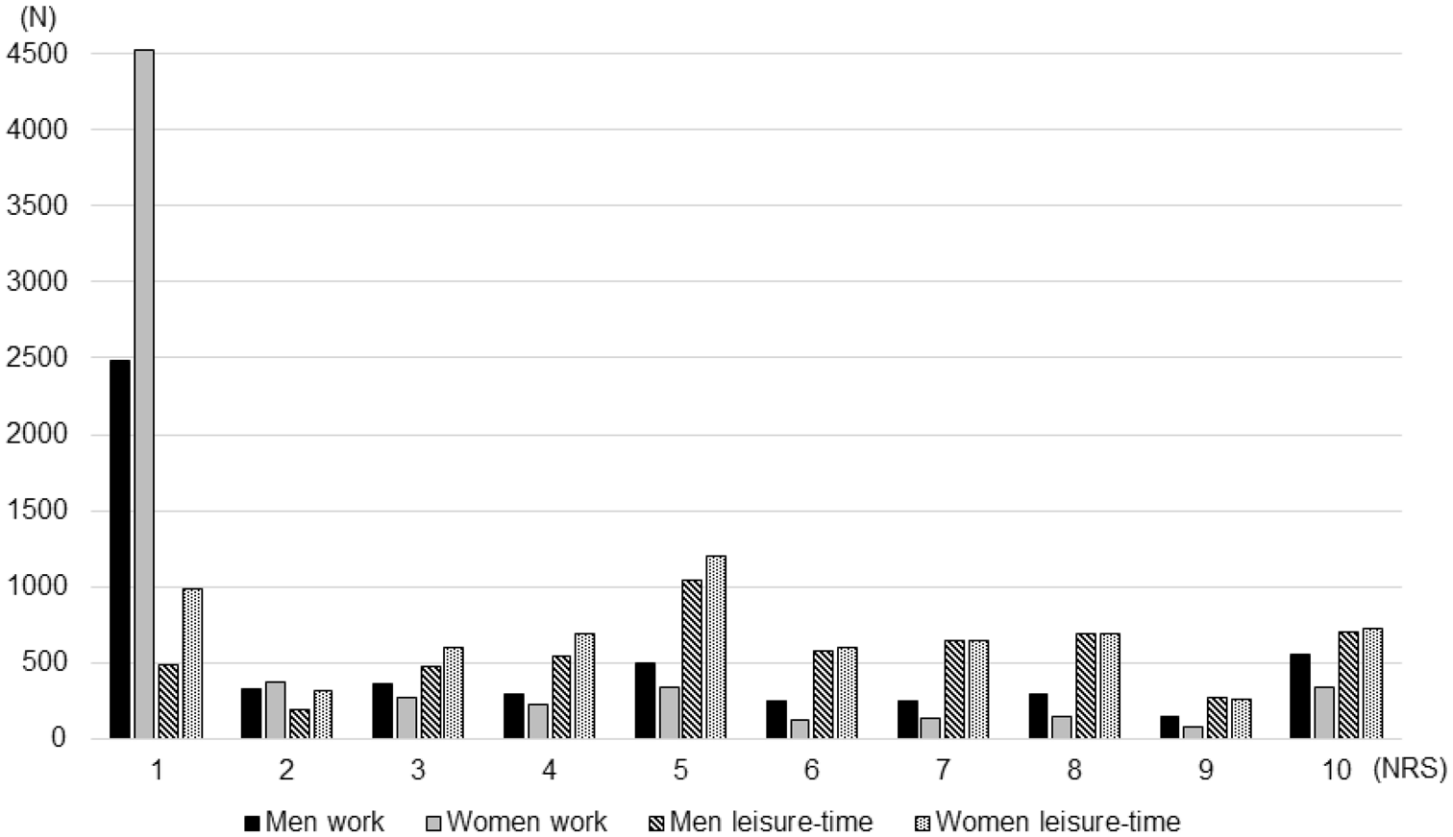
Exposure to outdoor or cold environments, categorized by work or leisure time, and gender. Cold exposure on the numerical rating scales (NRS) is presented on the *x* axis, and number of subjects on the *y* axis

Among responders, 6886 (54.5%) were females, 1070 (8.6%) were daily smokers, and 1891 (15.2%) daily snuff users. Smokers reported a median consumption of ten cigarettes per day (IQR 9), and snuff users a median of 3 boxes per week (IQR 3). The median age was 54 years (IQR 23; range 18–70). Regarding current occupation, 2044 (16.8%) were professionals, 1831 (15.0%) service and sales workers, 1183 (9.7%) technicians and associate professionals, 1035 (8.5%) clerical support workers, 751 (6.2%) plant and machine operators and assemblers, 656 (5.4%) crafts and related trades workers, 461 (3.8%) managers, 297 (2.4%) manual workers, 233 (1.9%) self-employed, 217 (1.8%) skilled agricultural, forestry, and fishery workers, and 32 (0.3%) professional militaries. In addition, 2284 (18.8%) were retired, 678 (5.6%) students, 239 (2.0%) unemployed, 173 (1.4%) on sick leave, and 65 (0.5%) on parental leave. Regarding occupational cold exposure, 7013 (58.5%) reported the lowest alternative on the ten-degree scale, while leisure-time exposure was more evenly distributed (Fig. 1). The groups with the highest occupational cold exposure ratings on the ten-digit NRS scale were skilled agricultural, forestry, and fishery worker (median 9; IQR 4); professional militaries (median 8,5; IQR 3); and crafts and related trades workers (e.g. construction workers) (median 5; IQR 6).

In the survey, 2464 (19.8%) reported wheeze, 2769 (22.2%) chronic cough, and 2734 (22.0%) productive cough. A total of 1498 (12.1%) reported asthma, and of these 1137 (75.9%) had prescription treatment, and 821 (54.8%) reported worsening during cold spells. COPD was reported by 151 (1.2%), of which 100 (66.2%) had prescription treatment, and 62 (41.1%) experienced worsening during cold spells. Among those who negated having asthma or COPD (N = 10713), wheeze was reported by 1371 (12.8%), chronic cough by 2006 (18.7%), and productive cough by 1861 (17.4%). Additional descriptive data are available in Table 1.

**Table 1:** Descriptive characteristics of the study participants, separated by airway symptom category

Variable	Categories	Wheeze		Chronic cough		Productive cough		No airway symptoms	
		*N*	%	*N*	%	*N*	%	*N*	%
Gender	Male	1037	42.1	1143	41.3	1254	45.9	3632	46.1
	Female	1427	57.9	1626	58.7	1480	54.1	4241	53.9
Age group (years)	18–40	538	21.8	706	25.5	667	24.4	1963	24.9
	41–54	643	26.1	702	25.4	678	24.8	2148	27.3
	55–63	681	27.6	741	26.8	721	26.4	1883	23.9
	64–70	602	24.4	620	22.4	668	24.4	1879	23.9
Body mass index (kg/m^2^)	BMI < 18.5	30	1.2	45	1.7	46	1.7	83	1.1
	18.5 ≤ BMI < 25	743	30.9	1055	39.0	972	36.4	3677	47.7
	BMI ≥ 25	1628	67.8	1606	59.3	1650	61.8	3953	51.3
Geographical region	Alpine	584	23.7	575	20.8	620	22.7	1690	21.5
	Inland	668	27.1	707	25.5	718	26.3	2055	26.1
	Coastal	1212	49.2	1487	53.7	1396	51.1	4128	52.4
Current work status	Working	1668	69.7	1883	70.1	1801	68.5	5539	72.7
	Not working^a^	724	30.3	802	29.9	830	31.5	2084	27.3
Tobacco habits	Daily smoking	395	16.1	358	13.0	412	15.2	465	6.0
	Daily snuff use	431	17.6	425	15.4	487	17.9	1102	14.1
Previous diseases	Asthma	937	39.3	634	23.5	720	27.0	349	4.5
	COPD	119	4.9	88	3.2	115	4.3	16	0.2
	Hypertension	773	32.1	777	28.5	790	29.5	1671	21.6
	Diabetes mellitus	163	6.7	170	6.2	175	6.5	354	4.5
	Angina pectoris	107	4.4	95	3.5	113	4.2	135	1.7
	Myocardial infarction	92	3.8	85	3.1	101	3.8	140	1.8
	Stroke	55	2.3	59	2.2	64	2.4	113	1.5
Occupational cold exposure	Any (NRS > 1)	1026	43.6	1155	43.4	1161	44.7	3038	40.4
	None (NRS 1)	1329	56.4	1504	56.6	1438	55.3	4478	59.6
Leisure time cold exposure	High (NRS > 5)	1136	47.0	1277	46.8	1242	46.3	3636	47.0
	Low (NRS ≤ 5)	1282	53.0	1449	53.2	1438	53.7	4094	53.0

*BMI* body mass index, *COPD* chronic obstructive pulmonary disease, *NRS* numerical rating scale

^a^Pensioners, students, unemployed, and those on sick or parental leave

### Binary logistic regression analyses

**Fig. 2:**
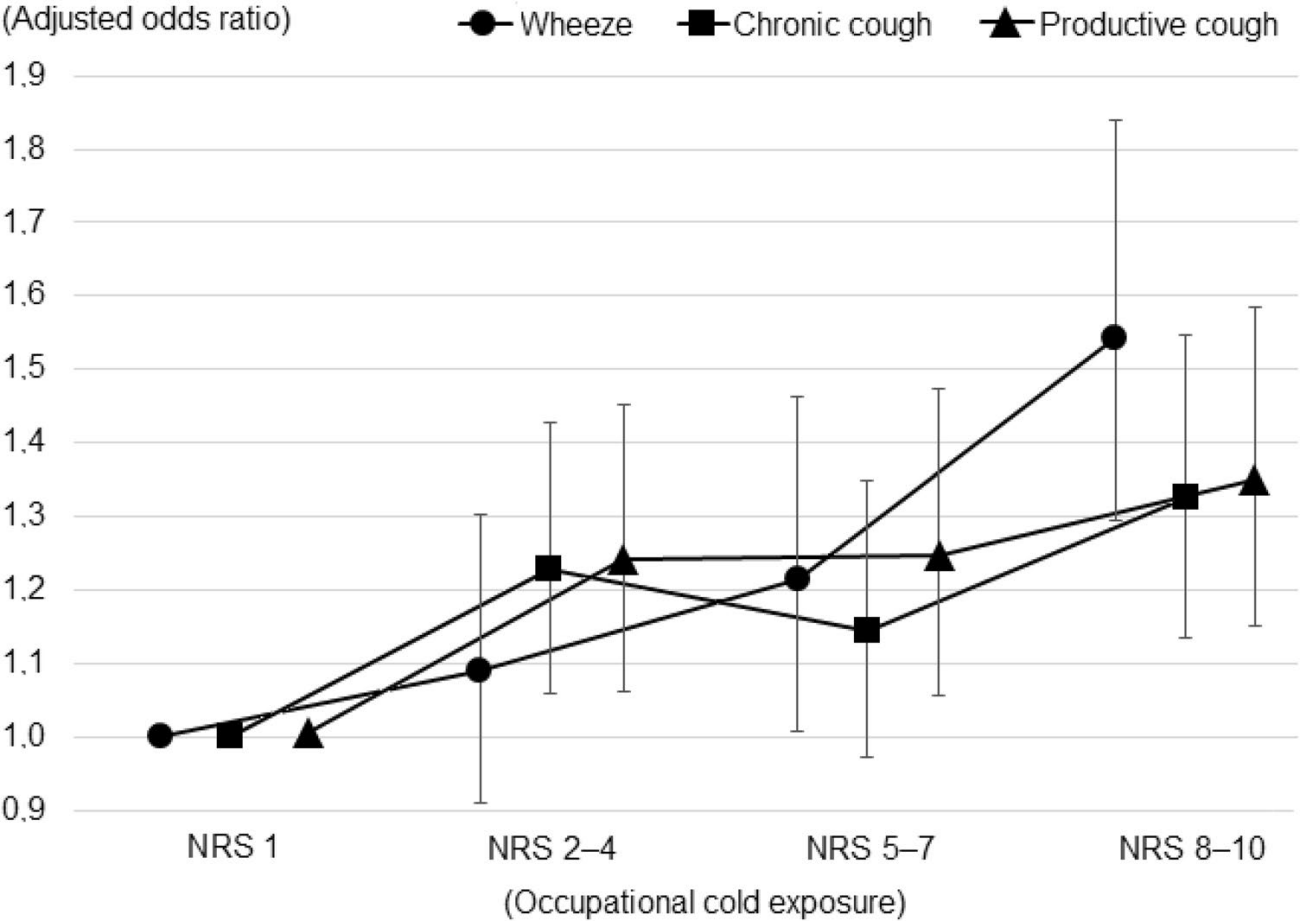
Adjusted odds ratios for reporting wheeze, chronic cough, or productive cough in relation to occupational cold exposure, categorized into four groups based on the responses on the numerical rating scale (NRS) for occupational cold exposure, among currently working subjects (*N* = 8740). Odds ratios are adjusted for gender, age, body mass index, daily smoking, asthma, and chronic obstructive pulmonary disease. Error bars depict the 95% confidence intervals

The crude OR (95% CI) for all subjects (*N* = 12627) between reporting any occupational cold exposure (above the 50th percentile, NRS > 1) and wheeze was 1.1 (1.0–1.2); for chronic cough 1.1 (1.0–1.2); and for productive cough 1.2 (1.1–1.3). After excluding subjects not currently working (pensioners, students, unemployed, and those on sick or parental leave; *N* = 3439), as well as those who had not specified their occupation (*N* = 448), and adjusting for relevant covariates (gender, age, BMI, daily smoking, asthma, and COPD), the results were 1.3 (1.1–1.4); 1.2 (1.1–1.4); and 1.3 (1.1–1.4), respectively (Table 2). Subsequent division of occupational cold exposure into four categories revealed a dose–effect pattern (Fig. 2). A complete regression table for the final model is also available (Online Resource 1).

**Table 2:** Odds ratios (OR) and 95% confidence intervals (95% CI) for the association between reporting any occupational cold exposure and wheeze, chronic cough, or productive cough

Any occupational cold exposure (NRS > 1)	Wheeze	Chronic cough	Productive cough
	OR (95% CI)	OR (95% CI)	OR (95% CI)
Crude	1.1 (1.0–1.2)*	1.1 (1.0–1.2)*	1.2 (1.1–1.3)*
Restricted	1.2 (1.1–1.4)*	1.2 (1.1–1.3)*	1.3 (1.2–1.4)*
Model 1	1.3 (1.2–1.5)*	1.3 (1.1–1.4)*	1.3 (1.2–1.5)*
Model 2	1.3 (1.1–1.4)*	1.3 (1.1–1.4)*	1.3 (1.1–1.4)*
Model 3	1.3 (1.1–1.4)*	1.2 (1.1–1.4)*	1.3 (1.1–1.4)*

*Significant at the 0.05 level. Crude: All subjects (*N* = 12627); Restricted: Only currently working subjects (*N* = 8740); Model 1: Restricted group adjusted for gender, age and body mass index; Model 2: Model 1 adjusted for daily smoking; Model 3: Model 2 adjusted for asthma and chronic obstructive pulmonary disease

The corresponding crude OR for all subjects (*N* = 12627) between reporting leisure-time cold exposure (above the 50th percentile, NRS > 5) and wheeze was 1.0 (95% CI 0.9–1.1); for chronic cough 1.0 (95% CI 0.9–1.1); and for productive cough 1.0 (0.9–1.1). Multiple modeling adjusting for all of the factors listed above yielded odds ratios of 1.1 (95% CI 1.0–1.2); 1.0 (0.9–1.1); and 1.0 (0.9–1.1), respectively.

## Discussion

### Main results

This epidemiological study, conducted on a sample of the general population in northern Sweden, showed that occupational cold exposure was associated to reporting wheeze (OR 1.3; 95% CI 1.1–1.4); chronic cough (OR 1.2; 95% CI 1.1–1.4); and productive cough (OR 1.3; 95% CI 1.1–1.4), after adjusting for gender, age, body mass index, smoking, asthma and COPD. Leisure-time cold exposure was not a significant predictor of airway symptoms.

### Interpretation

The results of the present study are in line with other epidemiological studies showing increased airway morbidity from cold air exposure (Analitis et al. 2008; Hajat et al. 2007; Harju et al. 2010; Kotaniemi et al. 2002; Mäkinen and Hassi 2009; Millqvist et al. 1987; Näyhä 2005; Schwartz 2005; The Eurowinter Group 1997), as well as studies with an occupational perspective on cold-related airway symptoms (Jammes et al. 2002; Kotaniemi et al. 2003; Reijula et al. 1990). The prevalence of self-reported physician-diagnosed asthma in the present study was 12.1%. This can be compared to other recent surveys on the general population, reporting a prevalence of 9.0% among subjects aged 25–74 in northern Finland (Harju et al. 2010), and 10.9% among subjects aged 20–69 years in northern Sweden (Backman et al. 2017). For COPD, the self-reported prevalence was 1.2% in the present study, compared to 2.7% in the aforementioned finish study (Harju et al. 2010), and 2.9% in finish Lapland (Kotaniemi et al. 2002), while a spirometry study in northern Sweden reported moderate to severe COPD in 3.9% of subjects aged 23–72 (Backman et al. 2016). This span in prevalence is likely dependent upon differences in age distribution, smoking habits, and subject sampling techniques. A spirometrically defined diagnosis is also likely to be more sensitive than survey-based definitions, since spirometry can reveal mild obstructive lung disease in asymptomatic individuals (Lindberg et al. 2006).

The prevalence of wheeze, chronic cough and productive cough was roughly similar (19.8–22.2%) in the present study, as was the effect size (OR 1.2–1.3) of occupational cold exposure on reporting these outcomes (Table 2). In comparison, a large population-based survey in Finland reported an association between occupational cold exposure and shortness of breath (OR 1.2; 95% CI 1.0–1.5) and chronic bronchitis (OR 1.8; 95% CI 1.2–2.6), in multiple logistic regression modelling (Kotaniemi et al. 2003). Importantly, in the present study, airway symptoms were common also among those with no previous lung condition. The smoking prevalence in the present study (8.6%) was slightly lower than the 10.5–12.1% (depending on gender) that was reported for subjects living in northern Sweden in 2009 (Eriksson et al. 2011). This difference might reflect an ongoing decrease in habitual smoking over time, and possibly also an effect of the overrepresentation of subjects living in the coastal region in the present study (Table 1), where socioeconomic status is higher, and prevalence of smoking lower (data not shown).

In the multiple model, gender and age were adjusted for since previous studies have suggested that cold-related respiratory symptoms are more common among women, and generally increase with age (Kotaniemi et al. 2002; Mäkinen and Hassi 2009). BMI was thought of as an indicator of general health. Asthma and COPD were included since a previous study reported that cold-related airway symptoms were roughly twice as common among subjects with such conditions (Näyhä et al. 2011). Finally, smoking was included since it has been suggested to increase cold-related airway symptoms (Harju et al. 2010; Kotaniemi et al. 2002), and a dose–effect pattern between cigarette consumption and airway symptom frequency was noted the present study (data not shown). Adjusting for all these factors only had a minor effect, which supports the notion that occupational cold exposure can be considered a robust and independent predictor of airway symptoms.

The hazardous potential of exposure to cold environments is likely dependent on ambient temperature, air humidity level, and exposure duration. The effects of cold climate are subsequently modified by individual factors, of which the degree of physical activity is likely the most important, since physical strain induces a higher respiratory rate, and a shift to mouth breathing, which increases the exposure of the airways to cold and dry air (Koskela 2007; McFadden et al. 1985). In fact, experimental studies show that high exercise intensity in a cold climate can induce lung function reduction in healthy individuals (Hanstock et al. 2020). The use of personal protection equipment, such as hooded jackets, and heat- and moisture-exchanging breathing devices, has been shown to alleviate exercise-induced asthma and other respiratory symptoms during physical activity in cold air (Frischhut et al. 2020; Jackson et al. 2020; Koskela 2007). Apart from health concerns, cold-induced respiratory problems can also decrease work performance, which is important from an employer perspective (Koskela et al. 1998; Mäkinen and Hassi 2009).

The reasons for occupational cold exposure being a stronger predictor for airway symptoms than leisure-time exposure are not known. Possible explanations could be that occupational exposure is generally of longer duration, demands a higher physical activity level, and cannot easily be refrained from during cold spells. For preventive purposes, it would be of great value to identify threshold temperatures, at which the risk of airway injury increases. Based on such thresholds, suitable temperature limits for work-related activities at different levels of physical activity could be established. Vulnerable groups, such as those with previous obstructive lung disease, could benefit from more restrictive limits. From a population-based perspective, temperatures ranging from − 7 to − 18 °C have been reported to provoke airway symptoms (Harju et al. 2010). For winter endurance sports, temperature limits between − 16 and − 25 °C have been used, but have proven insufficient to prevent airway symptoms (Hanstock et al. 2020). More research, with both laboratory and epidemiological approach, is needed to establish temperature thresholds for adverse respiratory health effects.

In Swedish workplace legislation (AFS 2020:1 provisions), it is stated that temporary outdoor workplaces should be designed so that the workers are protected from the weather. In the guidance section, it is suggested that an international standard (ISO 7730:2005) could be used to assess climatic influence. However, this ISO standard presents methods for predicting the general thermal perception, and degree of discomfort of people exposed to moderate thermal environments (10–30 °C), but not cold work. There is another standard (ISO 15743:2008) which addresses risk assessment and management of cold work, using both quantitative and non-quantitative methods. Since the present study adds to the growing body of data showing adverse health effects from occupational cold exposure, benefits in health and productivity could likely be gained from broadly implementing this standard. Finally, ISO 15265:2004 offers a standardized approach for occupational health care specialists in assessing physiological constraints during cold work, and could also prove useful in preventing cold-related airway disease.

### Limitations

One of the major limitations with the present study was the low response rate, which may have introduced a sampling bias, if non-response was not random with regard to independent and dependent variables. However, it has been argued that a large community-based recruitment may provide good generalizability even if the response rate is low, under the condition that sources of sampling bias other than non-response are limited (Blair and Zinkhan 2006). These include coverage bias, where a segment of the population is excluded (i.e. due to language barriers, illiteracy, or lack of postal address), and selection bias, where certain groups are given disproportionate chances of being selected to participate. There is no reason to believe that these two sources of sampling bias were a major issue in the present study. Another limitation is the cross-sectional nature of data, which cannot be used to establish causality. Furthermore, the self-reported cold exposure variables have not been validated against other means of exposure measurement, such as geographically stratified metrological data, or individually worn temperature loggers. The span of occupational cold exposure was considered suitable for dichotomization (none/any), since roughly half the population had reported the lowest alternative (Fig. 1), and the scale used was arbitrary and not easily converted to exposure duration or intensity. However, this approach assumes a step function of risk with homogeneity within groups, which has not been ascertained. This issue was addressed through a more detailed exposure categorization, which revealed a dose–effect pattern (Fig. 2). Another limitation was the self-reported health data. Diagnoses of asthma and COPD are not always clear-cut, and using medical records or spirometry could have improved the accuracy in prevalence estimates. Also, there was a large number of subjects currently not working (*N* = 3439; 28.2%), which may have attenuated the occupational perspective. Finally, there are likely other explanatory variables for airway symptoms that were not investigated in the present study, such as the presence of allergies, gastroesophageal reflux disease, vocal cord dysfunction, a family history of lung disease, or exposure to vapors, gases, dust and fumes.

### Strengths

However, the present study included more than 12000 subjects, and collected ample data on factors known to affect the reporting of airway symptoms. The resemblances in prevalence estimates to other recent Swedish studies (Backman et al. 2016, 2017; Eriksson et al. 2011) support that the study sample was indeed representative of the general population. The study was performed during the winter season, which lessened the risk of recall bias.

### Conclusions

Occupational cold exposure was an independent predictor of airway symptoms in northern Sweden. Therefore, a structured risk assessment regarding cold exposure could be considered for inclusion in the Swedish workplace legislation.

## Supplementary Information

Below is the link to the electronic supplementary material.

Supplementary file1 (PDF 308 kb)

## Data Availability

Source data can be made available upon personal request.
