# Supplementary material for: Occupational cold exposure is associated with increased reporting of airway symptoms
Source: Int Arch Occup Environ Health. 2021 Apr 17;94(8):1945–52. doi: 10.1007/s00420-021-01694-y (PMC8490236; doi:10.1007/s00420-021-01694-y)
Supplement: Supplementary file 1 — Supplementary file1 (PDF 308 kb) [file 420_2021_1694_MOESM1_ESM.pdf]

Article title: Occupational cold exposure is associated with increased reporting of airway symptoms

Journal name: International Archives of Occupational and Environmental Health

Author names: Albin Stjernbrandt[1], Nikolai Stenfors[2], Ingrid Liljelind[1]

Affiliations: [1]Section of Sustainable Health, Department of Public Health and Clinical Medicine, Umeå University; [2]Section of Medicine, Department of Public Health and Clinical Medicine, Umeå University, Sweden

Corresponding author: Albin Stjernbrandt (albin.stjernbrandt@umu.se)

Online Resource 1. Complete logistic regression table (Model 3) showing crude and adjusted odds ratios and ninety-five percent confidence intervals for reporting wheeze, chronic cough, and productive cough among currently working subjects (N=8 740).

Variable	Categories	Wheeze<br>Crude<br>OR (95% CI)	Adjusted [a]<br>OR (95% CI)	Chronic cough<br>Crude<br>OR (95% CI)	Adjusted [a]<br>OR (95% CI)	Productive cough<br>Crude<br>OR (95% CI)	Adjusted [a]<br>OR (95% CI)
Occupational cold exposure	None (NRS≤1)	1.00	1.00	1.00	1.00	1.00	1.00
	Any (NRS 2–10)	1.23 (1.10–1.37)*	1.27 (1.11–1.44)*	1.20 (1.08–1.33)*	1.23 (1.10–1.38)*	1.30 (1.17–1.44)*	1.27 (1.13–1.43)*
Gender	Male	1.00	1.00	1.00	1.00	1.00	1.00
	Female	1.16 (1.04–1.29)*	1.27 (1.11–1.45)*	1.22 (1.10–1.36)*	1.32 (1.18–1.49)*	0.94 (0.85–1.05)	0.99 (0.87–1.11)
Age group (years)	18–40	1.00	1.00	1.00	1.00	1.00	1.00
	41–54	1.05 (0.91–1.21)	1.06 (0.89–1.25)	0.93 (0.81–1.06)	0.90 (0.79–1.04)	0.94 (0.82–1.07)	0.93 (0.80–1.08)
	55–63	1.28 (1.11–1.48)*	1.30 (1.09–1.54)*	1.11 (0.97–1.27)	1.05 (0.91–1.22)	1.11 (0.97–1.28)	1.05 (0.90–1.22)
	64–70	1.06 (0.85–1.31)	1.10 (0.85–1.42)	0.85 (0.69–1.05)	0.77 (0.61–0.96)*	0.91 (0.74–1.12)	0.77 (0.61–0.98)*
Body mass index (kg/m²)	<18.5	1.41 (0.77–2.59)	1.37 (0.68–2.73)	1.66 (0.98–2.80)	1.49 (0.86–2.59)	2.05 (1.22–3.44)*	1.98 (1.14–3.44)*
	18.5 ≤ BMI < 25	1.00	1.00	1.00	1.00	1.00	1.00
	BMI ≥ 25	1.96 (1.75–2.19)*	1.96 (1.71–2.24)*	1.32 (1.19–1.47)*	1.30 (1.16–1.45)*	1.53 (1.37–1.70)*	1.41 (1.25–1.59)*
Daily smoking	No	1.00	1.00	1.00	1.00	1.00	1.00
	Yes	2.54 (2.14–3.01)*	3.39 (2.79–4.11)*	1.95 (1.64–2.32)*	1.99 (1.66–2.39)*	2.81 (2.37–3.32)*	3.12 (2.60–3.75)*
Asthma	No	1.00	1.00	1.00	1.00	1.00	1.00
	Yes	11.85 (10.27–13.68)*	13.23 (11.34–15.43)*	2.87 (2.50–3.29)*	2.78 (2.41–3.21)*	4.33 (3.78–4.96)*	4.52 (3.92–5.22)*
COPD	No	1.00	1.00	1.00	1.00	1.00	1.00
	Yes	12.40 (6.87–22.38)*	7.55 (3.73–15.25)*	6.99 (4.06–12.04)*	4.54 (2.50–8.27)*	16.66 (8.62–32.18)*	11.60 (5.56–24.20)*

*Significant at the 0.05 level. [a] Adjusted for all other covariates. BMI: Body mass index; COPD: Chronic obstructive pulmonary disease; NRS: Numerical rating scale.
